# Demethylase Inhibitor Fungicide Resistance in *Pyrenophora teres* f. sp. *teres* Associated with Target Site Modification and Inducible Overexpression of *Cyp51*

**DOI:** 10.3389/fmicb.2016.01279

**Published:** 2016-08-19

**Authors:** Wesley J. Mair, Weiwei Deng, Jonathan G. L. Mullins, Samuel West, Penghao Wang, Naghmeh Besharat, Simon R. Ellwood, Richard P. Oliver, Francisco J. Lopez-Ruiz

**Affiliations:** ^1^Department of Environment and Agriculture, Centre for Crop and Disease Management, Curtin UniversityBentley, WA, Australia; ^2^Institute of Life Science, School of Medicine, Swansea UniversitySwansea, UK; ^3^School of Veterinary and Life Sciences, Murdoch UniversityMurdoch, WA, Australia

**Keywords:** Cyp51, azole, DMI, resistance, net blotch, *Pyrenophora teres*, overexpression, mutation

## Abstract

*Pyrenophora teres* f. sp. *teres* is the cause of net form of net blotch (NFNB), an economically important foliar disease in barley (*Hordeum vulgare*). Net and spot forms of net blotch are widely controlled using site-specific systemic fungicides. Although resistance to succinate dehydrogenase inhibitors and quinone outside inhibitors has been addressed before in net blotches, mechanisms controlling demethylation inhibitor resistance have not yet been reported at the molecular level. Here we report the isolation of strains of NFNB in Australia since 2013 resistant to a range of demethylase inhibitor fungicides. *Cyp51A:KO103-A1*, an allele with the mutation F489L, corresponding to the archetype *F495I* in *Aspergillus fumigatus*, was only present in resistant strains and was correlated with resistance factors to various demethylase inhibitors ranging from 1.1 for epoxiconazole to 31.7 for prochloraz. Structural *in silico* modeling of the sensitive and resistant CYP51A proteins docked with different demethylase inhibitor fungicides showed how the interaction of F489L within the heme cavity produced a localized constriction of the region adjacent to the docking site that is predicted to result in lower binding affinities. Resistant strains also displayed enhanced induced expression of the two *Cyp51A* paralogs and of *Cyp51B* genes. While *Cyp51B* was found to be constitutively expressed in the absence of fungicide, *Cyp51A* was only detected at extremely low levels. Under fungicide induction, expression of *Cyp51B, Cyp51A2*, and *Cyp51A1* was shown to be 1.6-, 3,- and 5.3-fold higher, respectively in the resistant isolate compared to the wild type. These increased levels of expression were not supported by changes in the promoters of any of the three genes. The implications of these findings on demethylase inhibitor activity will require current net blotch management strategies to be reconsidered in order to avoid the development of further resistance and preserve the lifespan of fungicides in use.

## Introduction

*Pyrenophora teres* f. sp. *teres* (*Ptt*) (*P. teres* Drechsler; anamorph *Drechslera teres* [Sacc.] Shoem.) is a necrotrophic fungal pathogen and the cause of net form of net blotch (NFNB) that, together with spot form of net blotch (SFNB), is one of the most important diseases of barley (*Hordeum vulgare*). Worldwide, net blotches are commonly responsible for barley yield losses of 10–40%, and in some cases losses of up to 100% can occur (Liu et al., 2011). NFNB is a major disease of most barley growing regions including Europe (Leišova et al., 2005; Serenius et al., 2007; Ficsor et al., 2014), North America (Tekauz, 1990; Steffenson et al., 1991), and North Africa (Boungab et al., 2012). In Australia, the value of disease control for NFNB is estimated at $98 million annually with average direct costs of $19 million annually, making it the most significant necrotrophic barley disease after SFNB (Murray and Brennan, 2010). Along with cultural practices, the main control measures are the application of effective fungicides and the use of cultivars with genetic resistance. However, due to the lack of highly resistant cultivars, net blotch diseases are mainly controlled using fungicides (Sierotzki et al., 2007). Fungicides of the quinone outside inhibitor (QoI) and the succinate dehydrogenase inhibitor (SDHI) classes are used in the control of *Ptt*, especially in Europe, where mutations in the respective target genes cytochrome b and the succinate dehydrogenase complex (*SdhB, SdhC*, and *SdhD*) associated with resistance in *Ptt* have now been widely reported (Semar et al., 2007; Sierotzki et al., 2007; Rehfus et al., 2016). In Australia, the fungicides used against *Ptt* are predominantly of the azole or demethylase inhibitor (DMI) group (APVMA, 2016).

The DMIs are a group of site-specific systemic fungicides and are the most important compounds used for the control of fungal pathogens in both medicine and agriculture (Becher and Wirsel, 2012; Ishii and Hollomon, 2015). This fungicide class is comprised of a large number of structurally diverse compounds, all having in common the presence of a N-substituted five or six-membered heterocyclic ring (Hof, 2001). The target site of all DMIs is the enzyme CYP51, a cytochrome P450 sterol 14α-demethylase essential to the biosynthesis of fungal sterols (López-Ruiz et al., 2010). Ergosterol is the most common sterol in fungi, being a vital component of the fungal cell membrane and essential for fungal growth (Köllerm, 2003).

Many cases of resistance to DMI fungicides have been documented in phytopathogenic fungi (Délye et al., 1997; Erickson and Wilcox, 1997; Fraaije et al., 2007; Ghosoph et al., 2007; Omrane et al., 2015). Acquired resistance to DMIs has also been widely reported in fungal pathogens of humans (Kanafani and Perfect, 2008; Morio et al., 2010; Howard and Arendrup, 2011; Becher and Wirsel, 2012). In filamentous fungi there are three principle known mechanisms of resistance; (1) target site modification, where point mutations in the *Cyp51* gene result in amino acid substitutions altering the structure of the CYP51 protein, and thus reducing the binding affinity of the fungicide to the enzyme. Point mutations have been observed to cause varying levels of cross resistance to different DMIs (Cools and Fraaije, 2013); (2) overexpression of the target gene(s) *Cyp51*. Increased production of the CYP51 enzyme results in a general reduction of sensitivity across all the DMIs (Ishii and Hollomon, 2015); (3) increased efflux by overexpression of membrane-bound drug transporters, reducing the accumulation of fungicide at the target site. Enhanced efflux tends to result in a phenotype of broad-spectrum resistance across the DMIs and unrelated fungicide classes (Ishii and Hollomon, 2015).

Several species of Ascomycete fungi have been shown to carry multiple *Cyp51* paralogs, including *Aspergillus* spp., *Fusarium* spp., *Penicillium digitatum*, and *Rhynchosporium commune* (Becher et al., 2011; Hawkins et al., 2014). *Cyp51B* is carried by all ascomycetes. Some species also carry a paralog termed *Cyp51A* (Becher et al., 2011). In species with three *Cyp51* genes, the third *Cyp51* is either a duplicated copy of *Cyp51A* (as in *A. flavus* and *A. oryzae*) or of *Cyp51B* (as in *A. terreus*) or a unique paralog termed *Cyp51C* (in *Fusarium* spp.) (Becher et al., 2011). In *R. commune one* of the *Cyp51* paralogs is a pseudogenized duplication of *Cyp51A* termed *Cyp51A-p* (Hawkins et al., 2014).

In fungi with multiple *Cyp51* paralogs, *Cyp51*-mediated azole resistance is often associated with mutations in or overexpression of the *Cyp51A* gene (Fan et al., 2013; Brunner et al., 2015). For example, in *A. fumigatus* mutations correlated with DMI resistance have been found only in the *Cyp51A* paralog and not in *Cyp51B* (Becher et al., 2011). Resistance to DMIs in *A. fumigatus* is also mediated by overexpression of the *Cyp51A* paralog, which in combination with mutations in *Cyp51A* results in cross-resistance (Mellado et al., 2007). Similarly, overexpression of the *Cyp51A* gene but not *Cyp51B* has been demonstrated as a mechanism for azole resistance in *A. flavus, A. niger, A. parasiticus*, and *Magnaporthe oryzae* (Yan et al., 2011; Fan et al., 2013). In *P. digitatum*, azole resistance has been shown to result from the overexpression of both *Cyp51A* and *Cyp51B* (Sun et al., 2013). In *R. commune*, azole resistance is associated with the presence of *Cyp51A* (Hawkins et al., 2014). An analysis of *R. commune* isolates showed no correlation between *Cyp51B* sequence and azole sensitivity, so the authors hypothesized that the selection pressure of azoles is driving the observed accumulation of polymorphisms in the sequence of *Cyp51A* (Brunner et al., 2015).

Resistance to the DMI fungicide triadimenol was first reported in *P. teres* isolates from New Zealand (Sheridan et al., 1985). Subsequent studies showed this phenotype emerged in both New Zealand and the United Kingdom from the early 1980s onwards (Sheridan et al., 1987). Crossing studies determined that in *P. teres* this resistance segregated to a single major genetic locus (Peever and Milgroom, 1992), and that resistance to other DMIs, including propiconazole, imazalil and fenarimol, was also genetically correlated (Peever and Milgroom, 1993). These studies did not distinguish the net and spot types of *P. teres*, although subsequent phylogenetic analysis has shown that the two *formae speciales* are genetically isolated and should be considered as distinct species (Rau et al., 2007). In *Ptt* specifically, an increase in tolerance to the DMI prochloraz has also been observed in isolates from Finland (Serenius and Manninen, 2008).

Here we report the discovery in *P. teres* f. sp. *teres* of resistance to multiple DMIs. Resistance was correlated with two genetic changes. Resistant isolates carried a novel mutation in a copy of the *Cyp51A* gene. Structural analysis of the CYP51A target revealed a strong correlation between this mutation and the resistance levels detected. These isolates also displayed overexpression of both copies of the *Cyp51A* gene as well as overexpression of the *Cyp51B*.

## Materials and methods

### Fungal isolates

The *Ptt* isolates used in this study are listed in Table 1. Isolates from 1996 to 2003 were collected by the Department of Agriculture and Food, Western Australia (South Perth, Western Australia). Isolates from 2009 onwards were collected and isolated by the authors from a combination of especially designed bait trials (years 2013, 2014, and 2015) and sampling trips. Bait trials sown with the NFNB susceptible variety Hindmarsh were designed to work as a fungicide resistance early warning system. 2 × 4 m plots were sprayed with either 1 × or 2 × the maximum registered dose of the fungicides epoxiconazole, tebuconazole, prothioconazole, and azoxystrobin, at growth stages GS31 and GS39. Treatments were replicated three times. Leaf samples from bait trials were collected 7 days following to the second spray application.

**Table 1:** **Details and fungicide sensitivity phenotype of isolates used in this study**.

**Year**	**Location**	**Phenotype**	**Isolate(s)**
1996	Kalannie, WA	TEB^−^	9179
1996	Merredin, WA	TEB^−^	9193
1996	Badgingarra, WA	TEB^−^	9238
1996	Pithara, WA	TEB^−^	9241
1996	Esperance, WA	TEB^−^	9254
1996	Jerramungup, WA	TEB^−^	9264
2003	Wongan Hills, WA	TEB^−^	10914
2009	Wongan Hills, WA	TEB^−^	W1
2012	Amelup, WA	TEB^−^	U9
2013	^a^Kojonup, WA	TEB^+^	Ko103, Ko309
2013	^a^Kojonup, WA	TEB^−^	Ko310, Ko603
2014	^a^Beverley, WA	TEB^+^	14P9FG30, 14P9FG32, 14P9FG34, 14P9FG40, 14P9FG43
2014	Kendenup, WA	TEB^−^	14P9FG430, 14P9FG435
2014	Woogenellup, WA	TEB^−^	14P9FG431, 14P9FG433, 14P9FG436
2014	Porongurup, WA	TEB^−^	14P9FG432
2014	Tenterden, WA	TEB^−^	14P9FG434, 14P9FG437
2014	Mt Barker, WA	TEB^−^	14P9FG438
2014	Arthur River, WA	TEB^−^	14P9FG439
2014	Takaralup, WA	TEB^−^	14P9FG440
2015	0631546E 6186387N	TEB^−^	15FRG002, 15FRG003
2015	South Stirling, WA	TEB^−^	15FRG094, 15FRG095, 15FRG096, 15FRG097, 15FRG098
2015	Esperance, WA	TEB^−^	15FRG133, 15FRG134, 15FRG135, 15FRG136
2015	Bakers Hill, WA	TEB^+^	15FRG146
2015	Kojonup, WA	TEB^−^	15FRG153, 15FRG154, 15FRG155, 15FRG156, 15FRG162, 15FRG164, 15FRG167, 15FRG168
2015	Kendenup, WA	TEB^−^	15FRG161, 15FRG172, 15FRG182, 15FRG183, 15FRG184, 15FRG185, 15FRG186, 15FRG197, 15FRG198, 15FRG199, 15FRG212
2015	West Arthur, WA	TEB^+^	15FRG219
2015	Dandaragan, WA	TEB^+^	15FRG220
2015	Wickepin, WA	TEB^−^	15FRG222
2015	Tenterden, WA	TEB^−^	15FRG223
2015	^a^Freeling, SA	TEB^−^	15FRG252, 15FRG253, 15FRG254, 15FRG255, 15FRG256
2015	^a^Williams, WA	TEB^−^	15FRG275, 15FRG276

Location: WA, Western Australia; SA, South Australia. Phenotype: TEB^^−^^, tebuconazole-sensitive; TEB^^+^^, tebuconazole-resistant.

a Bait trial.

Lesions were excised from dried leaf samples and surface sterilized for 30 s in 70% (v/v) ethanol, 60 s in 10% (v/v) NaOCl, and rinsed for 60 s in sterile deionized H_2_O. Surface-sterilized lesions were transferred to 1.5% (w/v) tap water agar plates amended with ampicillin (100 μg mL^−1^), streptomycin (100 μg mL^−1^), and neomycin (50 μg mL^−1^), and grown at room temperature for 7 days. Subsequent hyphal growth was sub-cultured to V8-potato-dextrose agar (10 g potato-dextrose agar, 3 g CaCO_3_, 15 g agar, 150 mL V8 juice in 850 mL sterile deionized H_2_O; V8PDA) plates and incubated at room temperature under white light for 5 days. To induce sporulation, sterile deionized H_2_O was applied to hyphae and flattened gently with a glass rod; the plate was then incubated at room temperature under near-UV for 24 h, followed by 15°C in darkness for 24 h. For each isolate, a single conidium was transferred to a new V8PDA plate. Mycelial plugs of this culture were stored at −80°C and these were used for all subsequent testing.

### *In vitro* fungicide sensitivity testing

Mycelial plugs of monoconidial *Ptt* isolates were inoculated on V8PDA plates and incubated at room temperature under white light for 5 days. Sporulation was induced as described above. Spores were harvested by flooding plates with 10 mL sterile deionized H_2_O and scraping the mycelia with glass rod. The suspension was then filtered through two layers of sterile cheesecloth and centrifuged at 3220 g for 10 min at 4°C. The pellet was resuspended in 1 mL sterile deionized H_2_O and spore density estimated using a haemocytometer. The suspension was adjusted to produce a stock with a spore concentration of 1 × 10^4^ mL^−1^.

Pure technical grade fungicide compounds were dissolved in absolute ethanol, and serial dilutions were prepared so that the total volume of fungicide/solvent added to each well was kept constant. A master mix was prepared consisting of 0.5% (v/v) fungicide stock, or an equivalent volume of ethanol (for the zero fungicide control), in Yeast Bacto Acetate liquid medium (10 g yeast extract, 10 g Bacto peptone, 10 g sodium acetate in 1 L sterile deionized H_2_O; YBA). A total volume of 95 μL was added to each well of a 96-well microtiter plate. The range of concentrations used for each fungicide was adapted to the response of each isolate to the fungicide tested in order to obtain a dose-response curve. In order to exclude the activity of alternative oxidase, salicylhydroxamic acid was included in the medium at a final concentration 50 μM for tests involving azoxystrobin.

A 5 μL volume of spore stock was inoculated to 95 μL of media with fungicide to a final concentration of 500 spores mL^−1^; at least two biological replicates with up to three technical replicates were inoculated for each isolate. Immediately following inoculation, optical density (OD) was measured at 405 nm wavelength in a Synergy HT microplate reader (BioTek). Plates were incubated for 96 h at room temperature in darkness, after which OD was again measured. Final OD values were normalized with readings taken immediately following inoculation. The concentration of fungicide resulting in 50% reduction in growth (EC_50_) was calculated in Microsoft Excel by linear regression of log_10_-transformed percentage reduction in OD compared to zero fungicide control, against log_10_-transformed concentration of fungicide. Estimate of EC_50_ was made with the linear portion of the dose response, using the formula $\text{E}{\text{C}}_{50}=10^{\left(\left[\text{log}10\left(50\right)-b\right]/m\right)}$, from the regression *y* = *m*.*log*(*concentration*)+*b*. The resistance factor (RF) of each resistant isolate was calculated as a ratio of the EC_50_ to the mean EC_50_ of all sensitive isolates collected in the years 1996 to 2012. All EC_50_ values were log_10_-transformed prior to statistical analysis (Liang et al., 2015), statistical analysis was performed with IBM SPSS (Statistical Product and Service Solutions, version 24.0, Armonk, NY, USA). EC_50_ values of populations were compared using Student's *t*-test with the significance threshold set at 5%; correlations between EC_50_ values were determined with Spearman's rank order correlation with the significance threshold set at 1%.

### Discriminatory dose screening

A 4 mm-diameter mycelial plug taken from the colony edge of a 5 day culture grown on V8PDA was inoculated to the center of a YBA agar plate amended with tebuconazole to a final concentration 10 μg mL^−1^, or equivalent volume of ethanol solvent for the zero fungicide control. Two biological replicates were inoculated for each isolate and plates were incubated for 96 h at room temperature in darkness, after which time the presence or absence of growth on the plate was visually assessed. Isolates able to grow at 10 μg mL^−1^ were considered to be resistant.

### Cloning and sequencing of *Cyp51A* and *Cyp51B* genes

Mycelia of *Ptt* was snap-frozen in liquid nitrogen and ground with tungsten beads in a Retsch MM301 Mixer-Mill (Retsch GmbH). Genomic DNA was isolated using a Biosprint 15 instrument and Biosprint DNA Plant Kit (QIAGEN) as per manufacturer's instructions. For sequencing of *Cyp51A* promoter, *Cyp51B* coding sequence and *Cyp51B* promoter, PCR amplification was carried out in 50 μL reaction volume containing 0.25 μL MyTaq DNA Polymerase (5 U μL^−1^, Bioline), 10.0 μL 5 × reaction buffer, 2.5 μL each forward and reverse primer (5 μM; Table 2), 2.5 μL DNA template (100 ng μL^−1^), and 32.25 μL H_2_O. PCR parameters were initial denaturation at 94°C for 2 min, followed by 35 cycles of 94°C for 30 s, 58°C for 30 s, and 72°C for 2 min, followed by a final extension at 72°C for 5 min. PCR products were purified with a GenElute PCR Clean-Up Kit (Sigma). Purified PCR products were sequenced by Macrogen (Seoul, South Korea). Sequences were assembled and analyzed using Geneious 6 software (Biomatters), alignments performed using ClustalW algorithm (Thompson et al., 1994) with IUB scoring matrix, gap opening penalty 15, gap extension penalty 6.66.

**Table 2:** **Oligonucleotide primers used in this study and their relevant characteristics**.

**Primer name**	**Sequence 5′–3′**	**Description**
PttCyp51A_1F	ATGCTCTCCCTCCTCTTCCTC	Forward primer for amplifying and sequencing *Cyp51A*
PttCyp51A_2F	TACGACTGATTGAGCAAGAGGT	Primer for sequencing *Cyp51A*
PttCyp51A_1R	GAGATCGTGGTACAGGCTTG	Primer for sequencing *Cyp51A*
PttCyp51A_3F	GCATTCCAACGTCGTCAAAG	Primer for sequencing *Cyp51A*
PttCyp51A_2R	TTCGCTGTTGGCTGAGATAC	Primer for sequencing *Cyp51A*
PttCyp51A_3R	TTACCGCCTCTCCCAGC	Reverse primer for amplifying and sequencing *Cyp51A*
PyrCyp51B_F1	AGTCGTCCACGCCTGTCG	Forward primer for amplifying and sequencing *Cyp51B*
PyrCyp51B_R1	TCTTGTGTGATGAGGGTGACG	Primer for sequencing *Cyp51B*
PyrCyp51B_F2	CATCACACAAGAATGCGAAGAC	Primer for sequencing *Cyp51B*
PyrCyp51B_F3	AGGAAACCCTCCGTATCCAC	Primer for sequencing *Cyp51B*
PyrCyp51B_R2	GAGTGTGTGGGAAGTGGGAAC	Primer for sequencing *Cyp51B*
PyrCyp51B_R3	CACTCAACTATGCCAGGTGCT	Reverse primer for amplifying and sequencing *Cyp51B*
PttCyp51A_Pro_F	GGCTCATAAATGGCGGAAC	Forward primer for amplifying and sequencing *Cyp51A* promoter
PttCyp51A_Pro_R	AGGAAGAGGAGGGAGAGCAT	Reverse primer for amplifying and sequencing *Cyp51A* promoter
PttCyp51B_Pro_F	CGTCAAGGGCAGCCGGATTA	Forward primer for amplifying and sequencing *Cyp51B* promoter
PttCyp51B_Pro_R	AGGCGTGGACGACTTGGATGTA	Reverse primer for amplifying and sequencing *Cyp51B* promoter
PttCyp51A_qPCR_F2	CGTGTACGACTGTCCCAATT	Forward primer for RT-qPCR of *Cyp51A*
PttCyp51A_qPCR_R2	TGCTCAATCAGTCGTACGTG	Reverse primer for RT-qPCR of *Cyp51A*
PttCyp51B_qPCR_F2	GGAGCAAACGTCCATCCTAG	Forward primer for RT-qPCR of *Cyp51B*
PttCyp51B_qPCR_R2	TGGATACGGAGGGTTTCCTT	Reverse primer for RT-qPCR of *Cyp51B*
PttActin_qPCR_F2	AATCGTCCGTGACATCAAGG	Forward primer for qPCR of *Actin*
PttActin_qPCR_R2	GTACGACTTCTCCAAGCTGG	Reverse primer for qPCR of *Actin*
WD001	TACTGTTCTACGCCCATCTCTC	Forward primer for copy number qPCR of *Cyp51A*
WD002	AATGCAGAGGGCGAGAAG	Reverse primer for copy number qPCR of *Cyp51A*

For cloning and sequencing of *Cyp51A* gene, PCR amplification was carried out in a 50 μL reaction volume containing 0.5 μL Phusion High Fidelity DNA Polymerase (2 U μL^−1^, Thermo Scientific), 10 μL 5 × reaction buffer, 1.5 μL DMSO, 5 μL dNTPs (2 mM), 2.5 μL each forward and reverse primer (10 μM; Table 2), 1 μL genomic DNA template (100 ng μL^−1^), 27 μL H_2_O. PCR parameters were initial denaturation at 98°C for 30 s, followed by 35 cycles of 98°C for 10 s, 69°C for 30 s, and 72°C for 1 min, followed by a final extension at 72°C for 10 min. PCR products were purified as described above. Purified PCR products were A-tailed with DyNAzyme II DNA Polymerase (2 U μL^−1^, Thermo Scientific), ligated into pGEM-T Easy Vector (Promega) as per manufacturer's instructions and transformed to XL10 Ultracompetent cells (Agilent). Plasmid DNA was prepared with a GenElute Plasmid Miniprep Kit (Sigma) and cloned inserts sequenced by Macrogen (Seoul, South Korea). Sequences were assembled and analyzed using Geneious 6 software (Biomatters), alignments performed using ClustalW algorithm (Thompson et al., 1994) with Blosum scoring matrix, gap opening penalty 10, gap extension penalty 0.5, free end gaps. Translated amino acid sequences were aligned to the archetypal CYP51A (Mair et al., 2016) amino acid sequence from *A. fumigatus* (GenBank accession no. AAK73659), and the CYP51B sequence from *P. digitatum* (GenBank accession no. ADO85402). Amino acid substitutions associated with resistance are described with reference to the archetype “mutation label” (italicized) throughout text as previously described (Mair et al., 2016). Sequences have been deposited to GenBank (accession numbers KX578217-KX578221).

### Growth of fungal cultures and nucleic acid extraction for quantitative PCR analysis and sequencing

Isolates Ko103 and 9193 were inoculated in 60 mL Fries liquid medium Number 2 (Fries, 1938) to a concentration of 500 spores mL^−1^ and grown at room temperature, 150 rpm in darkness with three biological replicates per isolate of both treatment and control cultures. Growth curve analysis of Ko103 and 9193 showed they had similar rates of growth (data not shown). At 64 h post-inoculation, when cultures were in the exponential phase of growth, tebuconazole in ethanol solution was added to the media to a final concentration equivalent to the EC_50_ of each respective isolate (EC_50_ = 3.9 μg mL^−1^ for Ko103; 0.26 μg mL^−1^ for 9193) as described previously (Cools et al., 2012). For the control cultures an equivalent volume of ethanol solvent was added with no tebuconazole. At 112 h post-inoculation, cultures were harvested and freeze-dried. RNA was extracted with Trizol reagent (Invitrogen), treated with RNase-Free DNase (QIAGEN), and purified with RNeasy Plant Mini Kit (QIAGEN). cDNA synthesis was performed with iScript cDNA Synthesis Kit (BioRad). Genomic DNA was extracted using the CTAB method (Saghai-Maroof et al., 1984). Concentration of nucleic acid was quantitated using a Quantus Fluorometer (Promega).

### Quantitative PCR analysis of gene expression and gene copy number

Quantitative RT-PCR (qPCR) analysis of the *Cyp51A* and *Cyp51B* genes was conducted with QuantiTech SYBR Green Mastermix (QIAGEN) on the BioRad CFX96 qPCR system using *Actin* as the endogenous control (NCBI accession no. XM_003298028) and primers listed in Table 2. All reactions were carried out with three biological replicates and three technical replicates each. Relative transcript abundances were calculated using the 2^−ΔCT^ method (Livak and Schmittgen, 2001). Statistical analysis was performed with IBM SPSS, relative expression ratios were compared using Mann–Whitney *U*-test with the significance threshold set at 5%. Gene copy number estimation by qPCR was performed as previously described (Solomon et al., 2008), using four biological replicates of genomic DNA and with *Actin* as the single copy control.

### Genome and RNA sequencing

All RNA sequencing and cDNA library construction was conducted by Novogene Bioinformatics Technology (Beijing, China). RNA was sequenced using Illumina HiSeq platform. Differential expression analysis was performed with edgeR (Robinson et al., 2010). Statistical analysis was performed with IBM SPSS, normalized read counts were compared using Student's *t*-test with the significance threshold set at 5%. Whole genome sequencing and library construction was performed by the Australian Genome Research Facility (Nedlands, Western Australia) using Illumina MiSeq platform. Raw Illumina reads were analyzed using FastQC (version 0.10.1; Andrews, 2010) for quality control. The filtered sequencing reads were assembled using SPAdes assembler (version 3.6.2; Nurk et al., 2013). Coverage cut-off was disabled and the number of mismatches and short indels was reduced by incurring SPAdes' build-in post processing module MismatchCorrector, which utilizes BWA tool (Li and Durbin, 2009). The obtained assembly was scaffolded using SSPACE (version 3.0; Boetzer et al., 2011). Annotation of the scaffolded assemblies was achieved by using Prokka tool (version 1.11; Seemann, 2014).

### *In silico* structural modeling

Structural modeling of CYP51A sensitive allele 9193-A1 and KO103-A1 allele carrying the F489L mutation was undertaken using an automated homology modeling platform as previously described for *Mycosphaerella graminicola* CYP51 and mutants (Mullins et al., 2011). Ligand docking of azoles was also carried out as previously described, and with AutoDock Vina (Trott and Olson, 2010), cross-referenced and corroborated using the heme-azole positions of the CYP51-azole co-crystallized structures of PDB:5EAB (*Saccharomyces cerevisiae* CYP51 complexed with S-tebuconazole) and PDB:5EAF (*Saccharomyces cerevisiae* CYP51 complexed with fluquinconazole).

## Results

### Identification of DMI fungicide resistance in *Ptt*

The fungicide sensitivities of monoconidial *Ptt* strains collected from 1996 to 2013 were determined *in vitro* via microtiter assay in order to establish EC_50_ levels to several DMIs as well as to fungicides from different mode of action groups. Isolates were screened initially with tebuconazole, epoxiconazole, and prothioconazole (Table 3), and a subset of these isolates tested against QoI fungicide azoxystrobin and the SDHI fungicide boscalid (Table S1). Two isolates, Ko103 and Ko309, were identified with reduced sensitivity to tebuconazole (EC_50_ of 3.9 and 2.4 μg mL^−1^, RF of 16.4 and 10.1, respectively) compared to the isolates collected during 1996–2012 (EC_50_ range 0.06–0.34 μg mL^−1^, mean EC_50_ = 0.24 μg mL^−1^). These two isolates also displayed a level of reduced sensitivity to other triazole fungicides, including epoxiconazole (EC_50_ of 0.23 and 0.16 μg mL^−1^, RF of 2.0 and 1.4, respectively) and prothioconazole (EC_50_ of 0.18 and 0.12 μg mL^−1^, RF of 2.7 and 1.6, respectively). By comparison, the EC_50_ values of the isolates collected during 1996–2012 were for epoxiconazole 0.02–0.23 μg mL^−1^ (mean EC_50_ = 0.11 μg mL^−1^) and for prothioconazole 0.03–0.21 μg mL^−1^ (mean EC_50_ = 0.07 μg mL^−1^). The analysis showed no significant differences in sensitivities of the DMI-resistant isolates to the non-DMI fungicides azoxystrobin [*t*_(10)_ = −0.62, *p* = 0.548] and boscalid [*t*_(9)_ = −0.65, *p* = 0.530], compared to the wild-type.

**Table 3:** **EC_50_ and RF values of *Pyrenophora teres* f. sp. *teres* isolates to tebuconazole, epoxiconazole, and prothioconazole**.

**Isolate**	**Tebuconazole**		**Epoxiconazole**		**Prothioconazole**	
	**EC_50_ (μg mL^−1^)**	**RF**	**EC_50_ (μg mL^−1^)**	**RF**	**EC_50_ (μg mL^−1^)**	**RF**
Ko103	3.9 (±0.9)^a^	16.4^b^	0.23 (±0.02)	2.0	0.18 (±0.04)	2.7
Ko309	2.4 (±0.2)	10.1	0.16 (±0.03)	1.4	0.12 (±0.01)	1.6
14P9FG30	4.1 (±0.5)	17.1	0.15 (±0.03)	1.3	0.25 (±0.04)	3.5
14P9FG32	3.8 (±0.5)	15.8	0.12 (±0.02)	1.1	0.38 (±0.08)	5.3
14P9FG34	4.8 (±0.6)	19.9	0.21 (±0.02)	1.8	0.17 (±0.02)	2.3
14P9FG40	4.2 (±0.3)	17.4	0.13 (±0.02)	1.1	0.11 (±0.01)	1.5
14P9FG43	3.3 (±0.8)	14.0	0.13 (±0.02)	1.1	0.12 (±0.02)	1.6
15FRG146	2.9 (±0.9)	12.0	0.23 (±0.06)	2.1	0.17 (±0.04)	2.3
15FRG219	5.3 (±0.7)	22.3	0.25 (±0.01)	2.2	0.18 (±0.03)	2.4
15FRG220	3.3 (±0.4)	13.8	0.16 (±0.02)	1.5	0.16 (±0.03)	2.2
		*M* = 16.5		*M* = 1.5		*M* = 2.6
9179	0.32 (±0.04)		0.21 (±0.04)		0.04 (±0.002)	
9238	0.15 (±0.03)		0.05 (±0.01)		0.03 (±0.004)	
9241	0.34 (±0.02)		0.17 (±0.01)		0.07 (±0.01)	
9264	0.11 (±0.03)		0.04 (±0.01)		0.03 (±0.003)	
10914	0.33 (±0.10)		0.23 (±0.04)		0.21 (±0.04)	
U9	0.06 (±0.01)		0.02 (±0.01)		0.05 (±0.02)	
Ko310	0.70 (±0.33)		0.17 (±0.04)		0.10 (±0.02)	
Ko603	0.44 (±0.08)		0.08 (±0.01)		0.10 (±0.03)	
9193	0.26 (±0.05)		0.12 (±0.02)		0.06 (±0.01)	
9254	0.28 (±0.05)		0.06 (±0.004)		0.03 (±0.01)	
W1	0.31 (±0.08)		ND^c^		ND	

EC_50_ values are the mean of at least two independent experiments.

a ± Standard error of the mean.

b Resistance Factor (EC_50_/mean EC_50_ of isolates 1996–2012).

c ND, Not determined.

The EC_50_ results from microtiter assays were used to define a cut-off concentration in order to distinguish the two tebuconazole sensitivity groups identified in the above screening. A total of 58 isolates collected in 2014 and 2015 were pre-screened on a discriminatory dose of 10 μg mL^−1^ tebuconazole (Table 1). Pre-screening identified a further eight isolates showing a reduced sensitivity to tebuconazole (Table 1). These eight new isolates were selected for more detailed analysis by EC_50_ screening on the previously tested DMI fungicides tebuconazole, epoxiconazole, and prothioconazole (Table 3). These isolates identified in pre-screening showed similar patterns of sensitivity to the original two resistant isolates Ko103 and Ko309, with RFs to tebuconazole of 12–19.9 (mean RF 16.5), to epoxiconazole 1.1–2.2 (mean RF 1.5), and to prothioconazole 1.5–5.3 (mean RF 2.6). The reduced sensitivity of the 10 isolates to DMIs was shown to be significant for tebuconazole [*t*_(12.56)_ = −12.32, *p* < 0.001], prothioconazole [*t*_(18)_ = −4.57, *p* < 0.001], and epoxiconazole [*t*_(10.99)_ = −2.41, *p* = 0.035], compared to the wild-types. There were significant positive correlations between the EC_50_ values of tebuconazole-prothioconazole [*r*_*S*(18)_ = 0.777, *p* < 0.001], tebuconazole-epoxiconazole [*r*_*S*(18)_ = 0.570, *p* = 0.009], and prothioconazole-epoxiconazole [*r*_*S*(18)_ = 0.593, *p* = 0.006].

These 10 tebuconazole-resistant isolates, and two tebuconazole-sensitive isolates, 9193 and 9254, were also tested for their sensitivity to two additional DMI compounds, propiconazole and metconazole (Table 4). The 10 tebuconazole-resistant isolates also showed reduced sensitivity to these DMIs compared to the two tebuconazole-sensitive isolates, with RFs to propiconazole of 5.7–9.7 (mean RF 7.7), and to metconazole 8.9–25.3 (mean RF 13.8). The two tebuconazole-sensitive isolates, 9193 and 9254, and six tebuconazole-resistant isolates (Ko103, Ko309, 14P9FG40, 14P9FG43, 15FRG146, and 15FRG220) were also tested for their sensitivity to a further four additional DMI compounds: triadimenol, triticonazole, difenoconazole, and prochloraz (Table 5). The six tebuconazole-resistant isolates also showed reduced sensitivity to these DMIs compared to the two tebuconazole-sensitive isolates, with RFs to triadimenol of 3.1–4.2 (mean RF 3.4), to triticonazole 10.8–19.1 (mean RF 15.6), to difenoconazole 10.2–15.2 (mean RF 12.4), and to prochloraz 17.8–31.7 (mean RF 27.7).

**Table 4:** **EC_50_ and RF values of *Pyrenophora teres* f. sp. *teres* isolates to metconazole and propiconazole**.

**Isolate**	**Metconazole**		**Propiconazole**	
	**EC_50_ (μg mL^−1^)**	**RF**	**EC_50_ (μg mL^−1^)**	**RF**
Ko103	1.8 (±0.5)^a^	11.4^b^	0.65 (±0.13)	7.1
Ko309	2.7 (±0.5)	16.7	0.51 (±0.08)	5.7
14P9FG30	2.4 (±0.6)	15.3	0.77 (±0.11)	8.5
14P9FG32	2.3 (±0.2)	14.8	0.74 (±0.12)	8.2
14P9FG34	1.4 (±0.2)	8.9	0.61 (±0.13)	6.7
14P9FG40	1.6 (±0.2)	10.0	0.70 (±0.08)	7.7
14P9FG43	1.7 (±0.3)	10.6	0.58 (±0.05)	6.4
15FRG146	1.9 (±0.4)	11.7	0.75 (±0.06)	8.2
15FRG219	4.0 (±0.5)	25.3	0.78 (±0.08)	8.5
15FRG220	2.2 (±0.3)	13.7	0.89 (±0.05)	9.7
		*M* = 13.8		*M* = 7.7
9193	0.11 (±0.02)		0.12 (±0.03)	
9254	0.21 (±0.05)		0.06 (±0.02)	

EC_50_ values are the mean of at least two independent experiments.

a ± Standard error of the mean.

b Resistance Factor (EC_50_/mean EC_50_ of isolates 1996–2012).

**Table 5:** **EC_50_ and RF values of *Pyrenophora teres* f. sp. *teres* isolates to triadimenol, triticonazole, difenoconazole, and prochloraz**.

**Isolate**	**Triadimenol**		**Triticonazole**		**Difenoconazole**		**Prochloraz**	
	**EC_50_ (μg mL^−1^)**	**RF**	**EC_50_ (μg mL^−1^)**	**RF**	**EC_50_ (μg mL^−1^)**	**RF**	**EC_50_ (μg mL^−1^)**	**RF**
Ko103	56.5 (±7.8)^a^	4.2^b^	26.3 (±6.0)	19.1	0.15 (±0.05)	15.2	0.29 (±0.03)	31.2
Ko309	51.7 (±7.1)	3.8	22.1 (±4.2)	16.0	0.10 (±0.05)	10.2	0.24 (±0.07)	25.7
14P9FG40	43.3 (±1.9)	3.2	18.3 (±2.7)	13.2	0.10 (±0.01)	10.5	0.28 (±0.04)	30.0
14P9FG43	41.1 (±6.6)	3.1	14.9 (±1.8)	10.8	0.10 (±0.03)	10.5	0.17 (±0.02)	17.8
15FRG146	41.1 (±7.0)	3.1	24.5 (±1.7)	17.7	0.14 (±0.03)	14.4	0.28 (±0.06)	30.0
15FRG220	43.2 (±3.6)	3.2	23.2 (±4.1)	16.8	0.14 (±0.02)	13.6	0.30 (±0.08)	31.7
		*M* = 3.4		*M* = 15.6		*M* = 12.4		*M* = 27.7
9193	21.1 (±2.1)		2.22 (±0.41)		0.011(±0.002)		0.008 (±0.001)	
9254	5.8 (±1.5)		0.54 (±0.02)		0.009 (±0.002)		0.011 (±0.002)	

EC_50_ values are the mean of at least two independent experiments.

a ± Standard error of the mean.

b Resistance Factor (EC_50_/mean EC_50_ of isolates 1996–2012).

### DMI resistance in *Ptt* is correlated with mutation F489L in one of the *Cyp51A* alleles

Sequencing of the 1.68 kb *Cyp51B* gene in all the tebuconazole-resistant isolates and in seven tebuconazole-sensitive isolates (W1, 9193, 9254, 9179, 9238, 9264, and 10914) showed the existence of a single allele of *Cyp51B* in all cases. There were no changes observed when the sequences were aligned to the *Cyp51B* reference of *Ptt* isolate 0–1 deposited in GenBank (accession no. NW_003523339).

**Figure 1:**
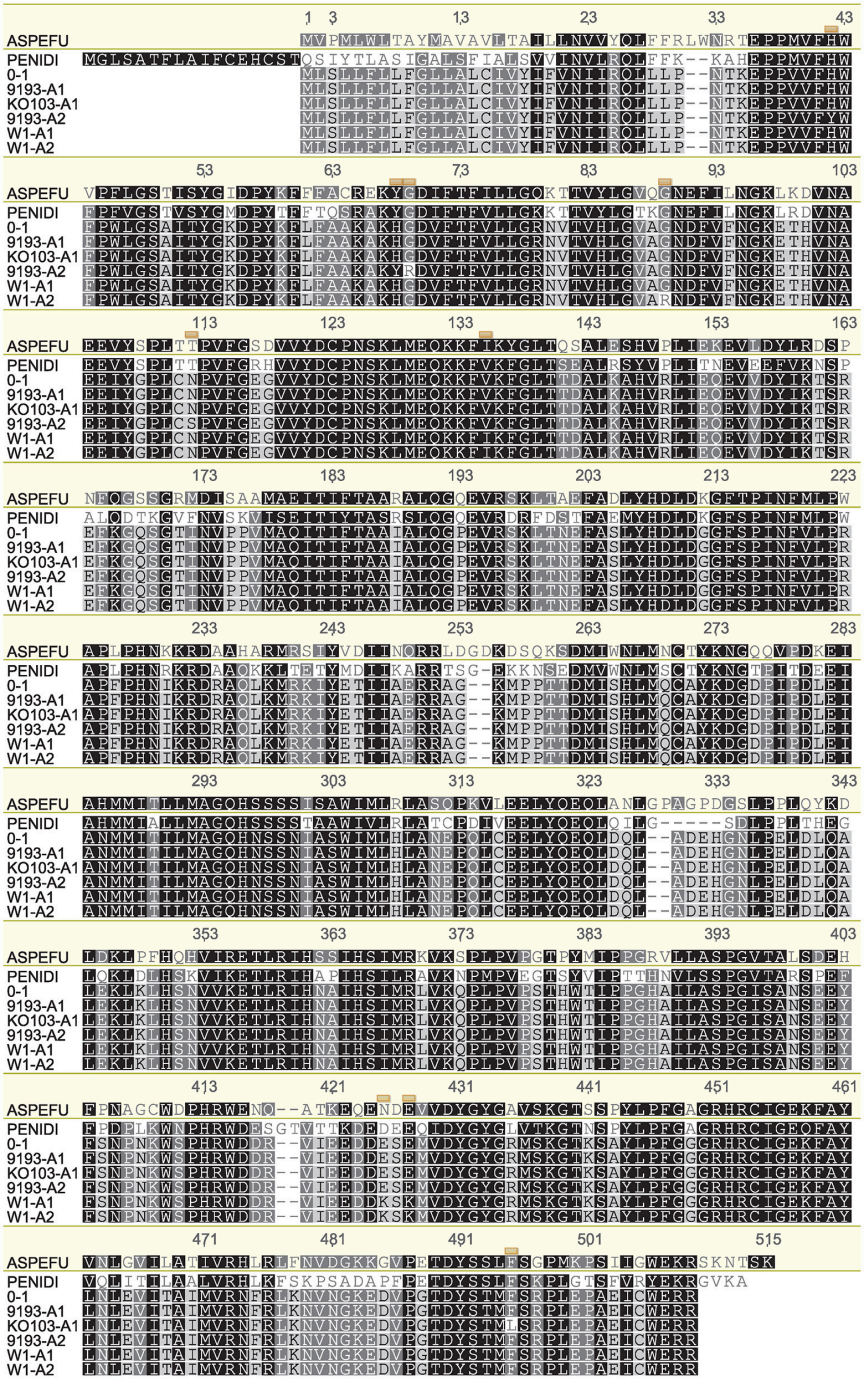
**Alignment of amino acid sequences corresponding to the different *Cyp51A* alleles**. Translated amino acid sequences of the five *P. teres* f. sp. *teres Cyp51A* alleles detected in this study (KO103-A1, 9193-A1, 9193-A2, W1-A1, W1-A2), aligned with CYP51A from the reference isolate 0–1 (GenBank accession no. XP_003303644), *Aspergillus fumigatus* CYP51A (ASPEFU, GenBank accession no. AAK73659), and *Penicillium digitatum* CYP51B (PENIDI, GenBank accession no. ADO85402). Alignment is numbered according to *A. fumigatus* CYP51A. The positions of polymorphisms in the *P. teres* f. sp. *teres* CYP51A sequences are indicated by yellow boxes. The amino acid sequence of 9193-A1 is identical to the 0–1 reference sequence. KO103-A1 differs from 9193-A1 by the amino acid substitution F489L, orthologous to F495I in *A. fumigatus* and F506I in *P. digitatum*. Alignment generated in Geneious version 6.1 software (Biomatters) using ClustalW algorithm with Blosum scoring matrix, gap opening penalty 10, gap extension penalty 0.5, free end gaps.

Genome sequencing of the isolates Ko103 and 9193 showed they both carried two copies of the *Cyp51A* gene (data not shown). This observation was later confirmed by qPCR (Figure S2). Subsequently, both copies of the *Cyp51A* gene were cloned and sequenced in 10 tebuconazole-resistant isolates and in three tebuconazole-sensitive isolates (W1, 9193, 9254), revealing the existence of five alleles of *Cyp51A*, assorted into three genotypes (Table 6). Sensitive isolates W1 and 9254 both carry the two alleles termed *W1-A1* and *W1-A2*, which differ by only one nucleotide at base 259, resulting in a substitution of arginine for glycine at position 87 of the translated amino acid sequence. The sensitive isolate 9193 carries the alleles termed *9193-A1* and *9193-A2*, with both of these alleles differing from the *W1-A1* and *W1-A2* alleles at four nucleotides including three non-synonymous changes, resulting in the point mutations V133I, K419E, and K421E in the amino acid sequences. The two 9193 alleles differ between each other by seven nucleotides including four non-synonymous changes, resulting in the point mutations H40Y, H66Y, G67R, and N110S in the amino acid sequence. The amino acid sequence of allele *9193-A1* is identical to that of the reference sequence of isolate 0–1 from GenBank (accession no. XP_003303644; Figure 1). All 10 resistant isolates also carry the *9193-A2* allele as well as a unique allele labeled *KO103-A1*. The *KO103-A1* allele is identical to the *9193-A1* allele except for a C to A transversion at base 1467 resulting in a substitution of leucine for phenylalanine at position 489 of the amino acid sequence. Thus, the F489L amino acid substitution represents the only polymorphism of *Cyp51A* segregating the resistant from the sensitive isolates. An alignment of the amino acid sequences of the CYP51A variants to the CYP51A amino acid sequence from *A. fumigatus* (Figure 1) revealed the F489L substitution to be orthologous to the archetype *F495I* mutation associated with DMI resistance in *A. fumigatus* and is therefore given the mutation label *F495L.* The *F495L* amino acid substitution is also homologous to the DMI resistance mutation F506I in *P. digitatum* CYP51B (Figure 1).

**Table 6:** **Translated amino acid sequences of *Cyp51A* alleles found in *Pyrenophora teres* f. sp. *teres***.

**Allele**		**Polymorphism in amino acid sequence**								**Found in isolate**
	**40**	**66**	**67**	**87**	**110**	**133**	**419**	**421**	**489**	
*W1-A1*	H	H	G	G	N	I	K	K	F	W1^a^, 9254^a^
*9193-A1*	H	H	G	G	N	V	E	E	F	9193^a^
*KO103-A1*	H	H	G	G	N	V	E	E	L	Ko103^b^, Ko309^b^, 14P9FG30^b^, 14P9FG32^b^, 14P9FG34^b^, 14P9FG40^b^, 14P9FG43^b^, 15FRG146^b^, 15FRG219^b^, 15FRG220^b^
*W1-A2*	H	H	G	R	N	I	K	K	F	W1, 9254
*9193-A2*	Y	Y	R	G	S	V	E	E	F	9193, Ko103, Ko309, 14P9FG30, 14P9FG32, 14P9FG34, 14P9FG40, 14P9FG43, 15FRG146, 15FRG219, 15FRG220

a Tebuconazole-sensitive isolate.

b Tebuconazole-resistant isolate.

### The *F495L* mutation in Cyp51A results in reduced binding affinity to azole fungicides

**Figure 2:**
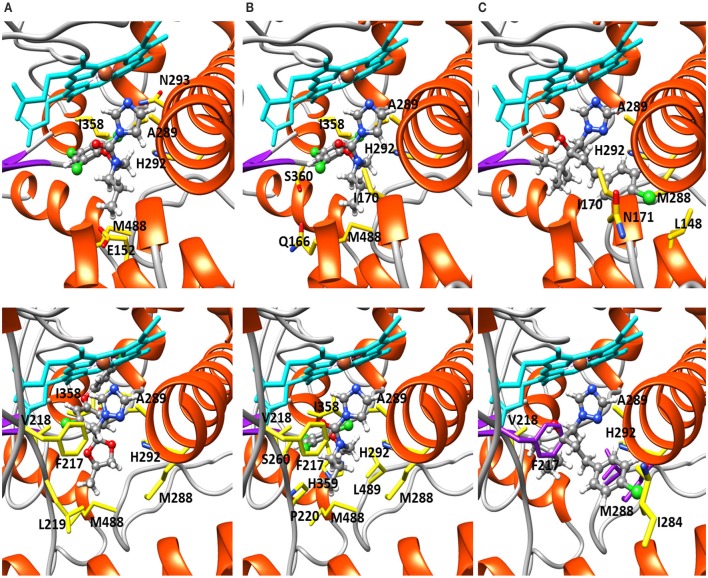
**Structural modeling of 9193-A1 and KO103-A1 docked with difenoconazole, prochloraz, and tebuconazole**. 9193-A1 (top row), KO103-A1 (bottom row). Fungicides in ball and stick, heme groups in cyan, showing the location of each azole and interacting residues shown in yellow and labeled. **(A)** Difenoconazole and **(B)** prochloraz occupy the binding cavities of 9193-1 and KO103-1 unhindered. **(C)** Tebuconazole binding is unhindered in 9193-1, but blocked by F217, M288, and L489 (in purple) in the KO103-1 mutant, as indicated by the spatial conflict. F217 is located away from the binding cavity in 9193-1.

Structural modeling was performed on the CYP51A variants 9193-A1 and KO103-A1 docked with the DMIs difenoconazole, prochloraz and tebuconazole (Table 7, Figure 2). The KO103-A1 allele with the *F495L* substitution had a lower predicted binding affinity for all three DMIs tested when compared to the 9193-A1 allele carrying *F495*. There were consistently more residues in close proximity to the docked azole compounds in KO103-A1 than in 9193-A1, suggesting a general diminishing of the binding cavity, but particularly markedly around the M288-H292 helical region. Residues A289 and H292 are consistently implicated to be in close proximity to the bound azole compounds in both 9193-A1 and KO103-A1, but in KO103-1 the close proximity is extended to M288 and G290.

**Table 7:** **Residues within 3 Å of the docked azoles and predicted binding affinities (kcal mol^−1^) of difenoconazole, prochloraz, and tebuconazole docked in 9193-A1 and KO103-A1**.

	**Difenoconazole**	**Prochloraz**	**Tebuconazole**
9193-A1	E152, **A289**^a^, **H292**, N293, N356, A357, I358	Q166, I170, **A289**, **H292**, A357, I358, H359, S360, M488, F489	L148, I170, N171, M288, **A289**, **H292**
Binding affinity	−10.600	−9.100	−9.500
KO103-A1	F217, V218, L219, **M288**, **A289**, **G290**, **H292**, N356, A357, I358, M488	F217, V218, P220, **M288**, **A289**, **G290**, **H292**, A357, I358, H359, S360, M488, L489	F217, V218, I284, **M288**, **A289**, **G290**, **H292**, L489
Binding affinity	−9.100	−7.500	−8.200^b^

a Residues in bold are part of the M288-H292 helical region of the binding cavity that are found in close proximity to the azole-heme complex.

b Predicted binding affinity based on alternative docking location.

The changes associated with CYP51A 9193-A1 do not prevent heme iron-coordinated binding of difenoconazole, prochloraz, or tebuconazole. In KO103-A1, however, there is substantial conformational change compared with 9193-A1, the foremost being movement of the long I helix at the amino terminal end around M288, leading to a constriction of the binding cavity; and the insertion of a section of beta turn, including F217 and V218, into the border of the heme cavity. In that regard, it is interesting to note that tebuconazole was predicted to dock adjacent to M288 in 9193-A1. The site of the primary mutation, F489, is itself within a 3 Å range of prochloraz in 9193-A1, and L489 (*F495L*) is again adjacent to prochloraz in KO103-A1 and is one of the three amino acids, along with F217 and M288 that prevents normal docking of tebuconazole.

### DMI resistance in *Ptt* is associated with overexpression of the *Cyp51A* and *Cyp51B* genes

**Figure 3:**
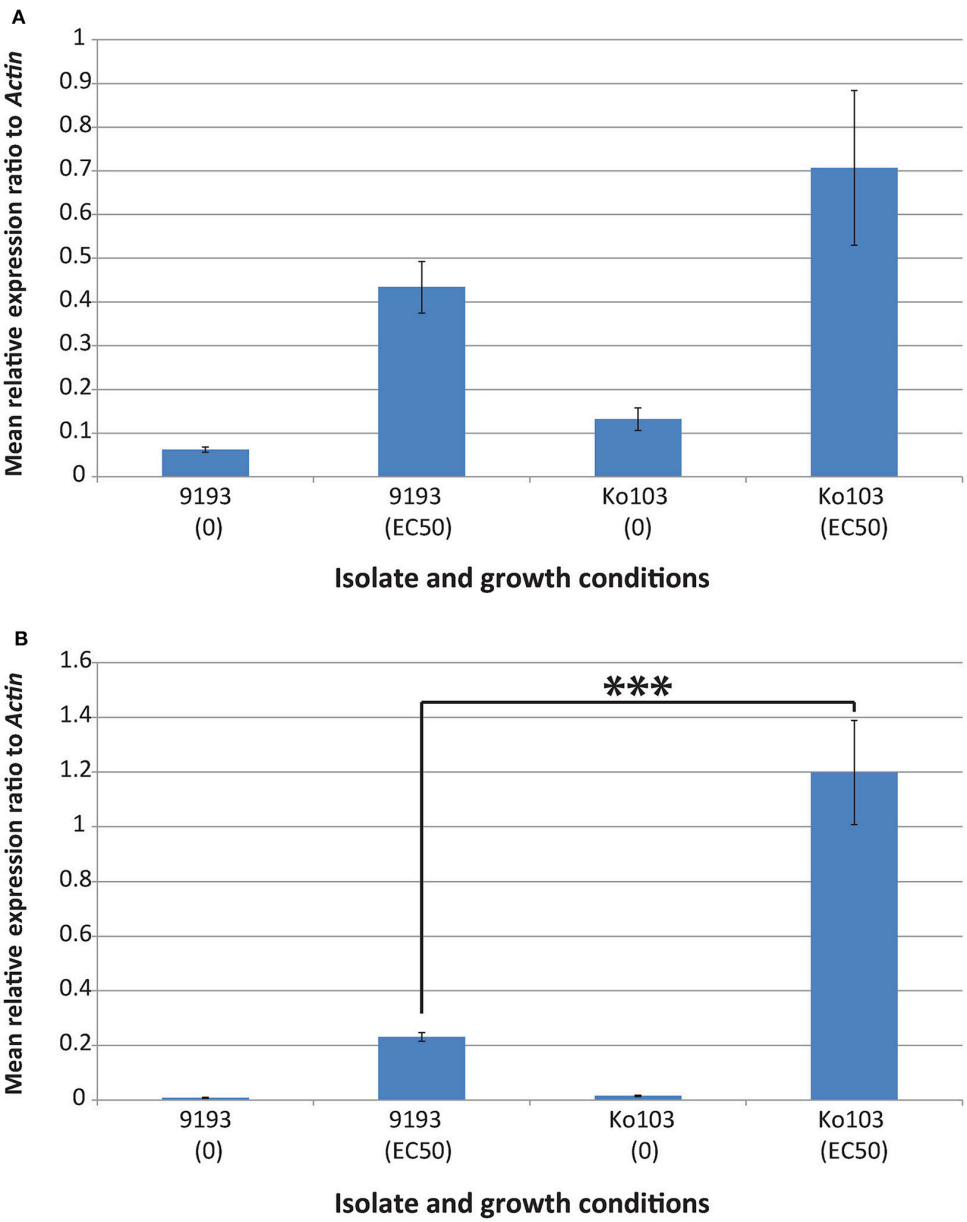
**Effect of tebuconazole on *Cyp51A* and *Cyp51B* gene expression in isolates 9193 and Ko103**. **(A)** qPCR confirmed constitutive expression of the *Cyp51B* gene in the ethanol control samples of both isolates. When grown under conditions that imposed a similar constraint on both isolates (EC_50_ tebuconazole), the expression of *Cyp51B* was found to be 1.6-fold higher in the resistant compared to the sensitive isolate. **(B)** qPCR showed *Cyp51A* (no distinction between alleles A1 and A2) to be expressed at extremely low levels in the control samples of either isolate. When grown under conditions that imposed a similar constraint on both isolates (EC_50_ tebuconazole), the expression of *Cyp51A* was 5.2-fold higher in the resistant compared to the sensitive isolate. *Cyp51A* expression appears to be induced by the presence of tebuconazole. Mean relative expression of genes of interest calculated by 2^−ΔCT^, normalized to *Actin* as the endogenous control (±Standard error of the mean, *n* = 3 biological replicates, three technical replicates per biological replicate). ^***^p < 0.001 (Mann–Whitney *U*-test).

**Figure 4:**
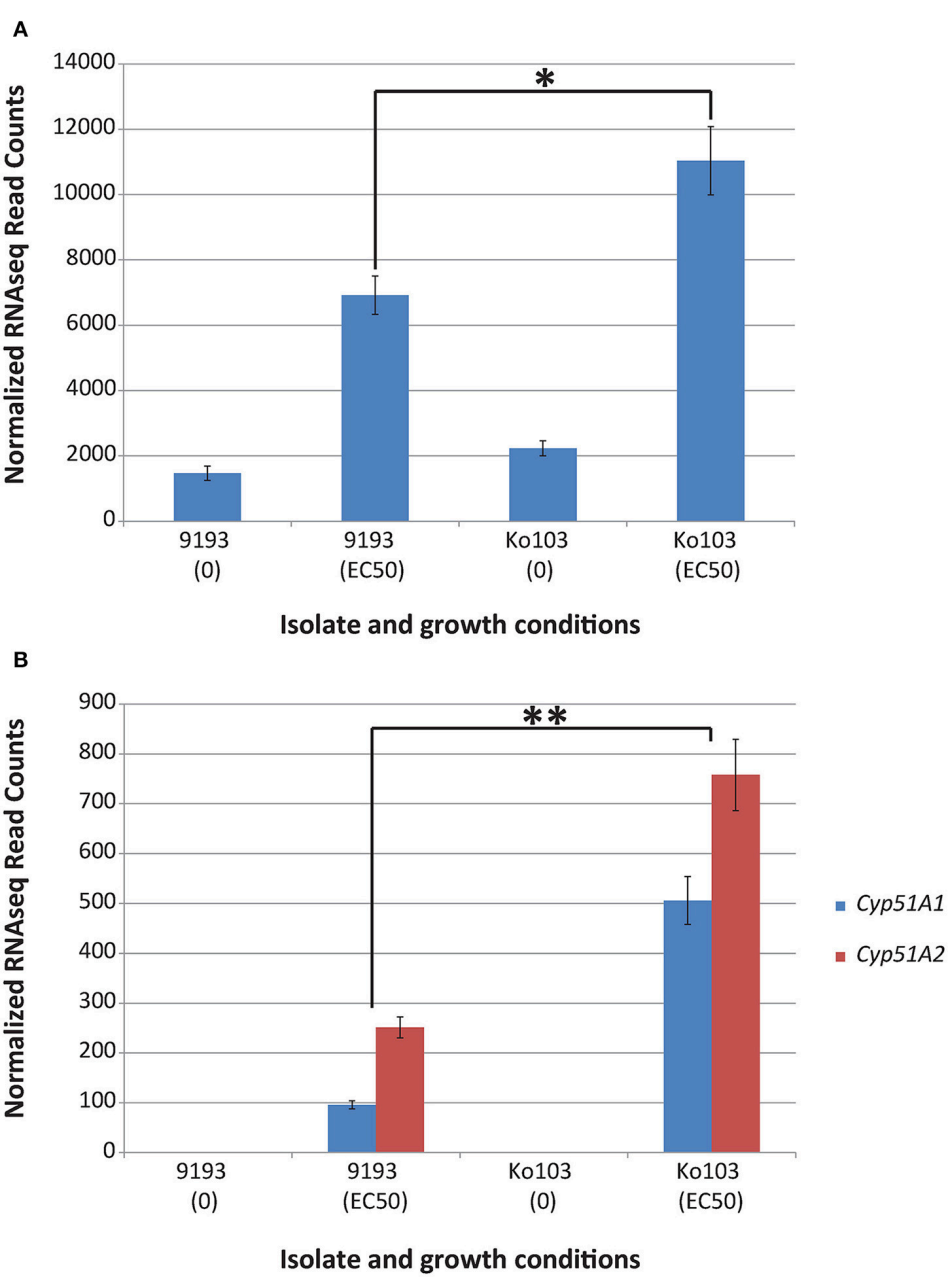
**RNAseq read counts of *Cyp51A1*, *Cyp51A2*, and *Cyp51B* in isolates 9193 and Ko103 under tebuconazole treatment**. **(A)** Mean RNAseq read counts of *Cyp51B*. Analysis of isolates Ko103 and 9193 showed the expression of *Cyp51B* to be 1.6-fold higher in the resistant compared to the sensitive isolate when grown under their corresponding EC_50_ tebuconazole. **(B)** Mean RNAseq read counts of *Cyp51A1* and *Cyp51A2*. Expression of *Cyp51A1* and *Cyp51A2* was not detectable (zero reads) in the control samples of either isolate. Read density analysis of RNA sequencing data of the two isolates confirmed that both copies of *Cyp51A* were expressed in both isolates. The expression levels of the *Cyp51A1* and *Cyp51A2* alleles in Ko103 and 9193 cultures amended with their corresponding EC_50_ tebuconazole, were shown to be 5.3- and 3-fold higher in Ko103 than in 9193, respectively. Differential expression analysis and read count normalization achieved by edgeR (±Standard error of the mean, *n* = 3 biological replicates). ^*^p < 0.05; ^**^p < 0.01 (Student's *t*-test).

To investigate the role of *Cyp51A* and *Cyp51B* expression on resistance, qPCR analysis and RNA sequencing of sensitive and resistant isolates induced with their respective EC_50_ tebuconazole concentrations were undertaken. Both experiments confirmed constitutive expression of the *Cyp51B* gene in the ethanol control samples (Figures 3A, 4A). By contrast, *Cyp51A1/A2* alleles were not detectable (RNA sequencing) or detected at extremely low levels (qPCR, no distinction between alleles *A1* and *A2*) in the control samples of either isolate (Figures 3B, 4B). Conditions that imposed a similar growth constraint on both isolates were used for studying the expression of *Cyp51A* and *Cyp51B* under fungicide induction (Cools et al., 2012). qPCR analysis comparing the tebuconazole-sensitive isolate 9193 and the resistant isolate Ko103 grown at their respective EC_50_ revealed the expression of *Cyp51B* to be 1.6-fold higher in the resistant compared to the sensitive isolate, although this difference was not considered significant (Mann–Whitney *U* = 33, *p* = 0.546; Figure 3A). The expression of *Cyp51A* was significantly higher in Ko103 than in 9193 (Mann–Whitney *U* = 0, *p* < 0.001), with a 5.2-fold increase in the resistant compared to the sensitive isolate (Figure 3B). These observations were validated by read density analysis of RNA sequencing data of the two isolates showing the expression of *Cyp51B* to be 1.6-fold higher in the resistant compared to the sensitive isolate at EC_50_ tebuconazole, which was considered significant {*t*_(4)_ = 3.44, *p* = 0.026, 95% CI [794, 7436]; Figure 4A}. The expression of the *Cyp51A* genes were on average 3.6-fold higher in the resistant compared to the sensitive isolate when grown at their respective tebuconazole EC_50_; RNA sequencing analysis also confirmed that both copies of *Cyp51A* were expressed in both isolates and further dissected the relative expression of the two *Cyp51A* copies (Figure 4B). The expression levels of the *Cyp51A1* and *Cyp51A2* alleles were shown to be 5.3-fold higher {*t*_(4)_ = 8.46, *p* = 0.001, 95% CI [275, 545]} and three-fold higher {*t*_(4)_ = 6.78, *p* = 0.002, 95% CI [299, 714]} in Ko103 than in 9193, respectively. The *Cyp51A2* gene was revealed to be expressed at a significantly higher level than the *Cyp51A1* gene in both isolate 9193 {2.6-fold higher, *t*_(4)_ = −6.84, *p* = 0.002, 95% CI [−219, −92.4]} and in Ko103 {1.5-fold higher, *t*_(4)_ = −2.92, *p* = 0.043, 95% CI [−491, −12.7]}. The results support the conclusion that *Cyp51A* expression appears to be induced by the presence of tebuconazole, especially in the resistant isolate.

### *Cyp51* gene overexpression in *Ptt* is not correlated with changes in the nucleotide sequences of the *Cyp51A* or *Cyp51B* promoters

Initially primers were used to amplify and sequence a 571 bp region upstream of the start codon of the *Cyp51B* gene in isolates 9193 and Ko103, revealing no changes at the nucleotide level between the two isolates. Subsequent genome sequencing of the two isolates allowed the investigation to be further extended to 1000 bp upstream of the start codon of the *Cyp51B* gene in isolates 9193 and Ko103, and also revealed no changes at the nucleotide level between the two isolates. The sequence data generated allowed the analysis of a region extending over 800 bp upstream of the start codons of both of the *Cyp51A1* and *A2* alleles, revealing that the sequence of the *Cyp51A1* and *A2* putative promoters are identical between isolates 9193 and Ko103 (Figure S3). However, comparing the promoter sequence of the *Cyp51A1* and *Cyp51A2* paralogs revealed several differences between them, including an 8 bp insert located 471 bp upstream of the start codon in the *Cyp51A2* promoter.

## Discussion

**Figure 5:**
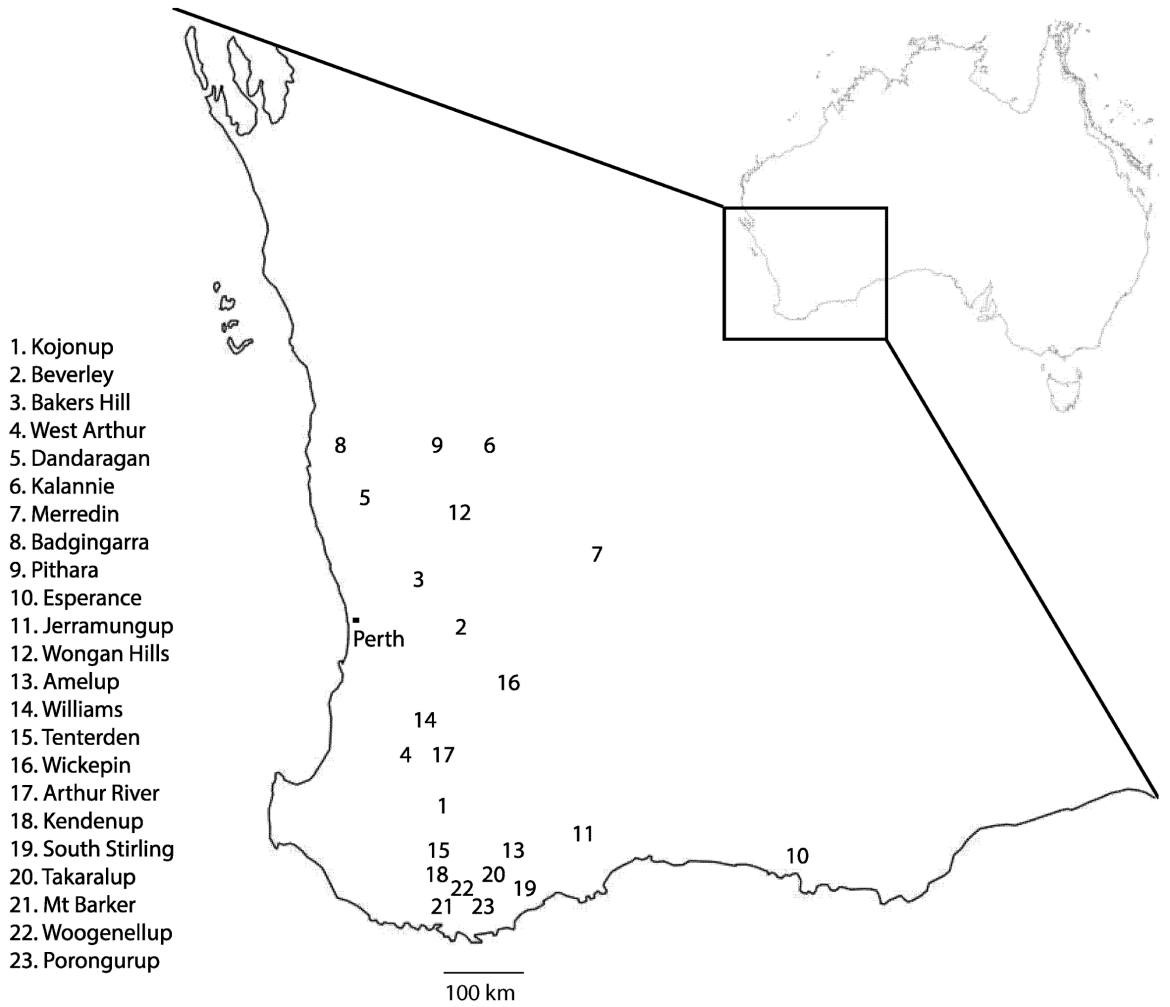
**Map of Western Australia showing geographic origin of *Pyrenophora teres* f. sp. *teres* isolates**. Leaf samples were collected from 23 separate locations in Western Australia from a combination of field trips and bait trials. Locations 1, 2, and 14 were specially designed bait trials. Resistant strains of *P. teres* f. sp. *teres* were isolated from samples taken in locations 1–5. Adapted from “Outline map of Australia” (http://www.ga.gov.au/metadata-gateway/metadata/record/gcat_61754) by Commonwealth of Australia (Geoscience Australia), used under CC BY 4.0.

In Australia, emergence of multi-DMI resistance in *Ptt* has been observed in isolates collected from the years 2013 onwards. Resistant isolates were geographically widely dispersed across Western Australia, originating in Kojonup, Beverley, Bakers Hill, West Arthur, and Dandaragan (Figure 5). We used bait trials in these studies so as to increase the likelihood of finding resistant isolates even when their frequency is still low. We therefore cannot reliably estimate the frequency of the resistant isolates, but it is clearly at a significant level in Western Australian populations of *Ptt* (Table 1).

Resistant isolates displayed high resistance factors (mean RF > 10) to tebuconazole, metconazole, triticonazole, difenoconazole, and prochloraz, and lower RFs (mean RF < 10) to epoxiconazole, prothioconazole, propiconazole, and triadimenol (Table 3). Isolates of this phenotype showed no observable reduction in sensitivity to QoI or SDHI fungicides. Although *Ptt* is a sexually recombining organism its reproduction occurs mostly asexually (Lehmensiek et al., 2010); therefore analysis of simple-sequence repeat markers conducted shortly after the emergence of a mutation should reveal whether the mutants are clonally related and derive from a single event, or whether this resistance has emerged multiple times independently as a response to the common selection pressure of azole fungicide use.

Two potential mechanisms of resistance have been observed in our data and may both contribute to the resistant phenotype. The observation that all DMI-resistant strains, and none of the DMI-sensitive strains, carry an identical point mutation resulting in the amino acid substitution *F495L* of the DMI target CYP51A, strongly supports the relationship of this mutation with azole fungicide resistance. The structural modeling and analysis of the CYP51A sensitive allele 9193-A1 and resistant allele KO103-A1 suggests that the *F495L* mutation has led to notable rearrangements of particular structural elements of the heme cavity, leading to localized constrictions, bringing more residues into proximity with all three of the azoles tested, most acutely in the region of the cavity adjacent to the docking site for the bulky less elongated tebuconazole, which provides a structural mechanism for the observed lack of sensitivity to azoles in isolates carrying the allele KO103-A1.

This conclusion is reinforced by an alignment of the *Ptt* CYP51A with its ortholog from *A*. *fumigatus*, showing that the F489L mutation is orthologous to *F495I* in *A*. *fumigatus* CYP51A. This mutation is associated with resistance to the DMI fungicides itraconazole and posaconazole in *A*. *fumigatus* (Mellado et al., 2007; Liu et al., 2015). Structural modeling of *A*. *fumigatus* CYP51A has shown that the *F495I* residue is close to the substrate binding pocket (Liu et al., 2016). The *F495L* mutation in *Ptt* and *A. fumigatus* CYP51A is also homologous to the F506I substitution in *P. digitatum* CYP51B, which is correlated with resistance to prochloraz (Wang et al., 2014). In *A*. *fumigatus* the *F495I* mutation has been found only in combination with other changes in the CYP51A amino acid sequence such as *L98H* or *S297T* (Mellado et al., 2007; Lockhart et al., 2011). In *P. digitatum*, the F506I substitution is found only in combination with G459S in CYP51B (Wang et al., 2014). By contrast, in *Ptt* the *F495L* mutation is the sole polymorphism of CYP51A to be found only in resistant and not in sensitive isolates.

Point mutations in the amino acid sequence of CYP51 targets occurring in conjunction with overexpression of the corresponding genes have been described in DMI-resistant strains of several pathogens. The predominant mechanisms of azole resistance in *A. fumigatus* is termed CYP51A TR/L98H, a combination of the amino acid substitution L98H with a 34 bp tandem repeat in the *Cyp51A* promoter that results in an up to eight-fold increase in expression (Mellado et al., 2007; Snelders et al., 2008; Lockhart et al., 2011). In *Pyrenopeziza brassicae*, the isolates with the highest levels of azole resistance have a combination of inducible *Cyp51B* overexpression and the amino acid substitution S508T (*S524T*) (Carter et al., 2014). In azole-resistant isolates of *Erysiphe necator*, the CYP51B Y136F (*Y137F*) amino acid substitution co-occurs with a 1.4 to 19-fold increase in expression (Rallos and Baudoin, 2016). In our study, induced overexpression of both the *Cyp51A* genes (*A1* and *A2*) and the *Cyp51B* gene has been demonstrated in a DMI-resistant strain compared to a DMI-sensitive wild-type and this may contribute to the observed phenotype of DMI cross-resistance. Alternatively, the observed increased expression of the *Cyp51* genes may be a compensatory mechanism to maintain sterol composition if the *F495L* mutation results in reduced CYP51A enzyme efficiency. Subsequent heterologous expression and enzymatic activity studies should determine the effect of the observed mutation on the function of CYP51A, and may clarify the role of target overexpression and dissect its contribution to DMI resistance.

Overexpression of *Cyp51* is typically caused by insertions of tandem repeats or transposable elements in the promoter region (Price et al., 2015). As described above, in *A. fumigatus* overexpression of *Cyp51A* is caused by a 34 bp tandem duplication in the promoter (Mellado et al., 2007). Similarly in *P. digitatum* a 126 bp tandem repeat acts as a transcriptional enhancer for *Cyp51A* expression in DMI-resistant isolates (Hamamoto et al., 2000). In *Pyrenopeziza brassicae* insertions of 46, 151, and 232 bp in the predicted promoter region of *Cyp51B* are correlated with overexpression (Carter et al., 2014). In azole-resistant isolates of *Zymoseptoria tritici*, overexpression of the *Cyp51B* gene is correlated with a 120 bp insertion in the predicted promoter region (Cools et al., 2012). In *Ptt* we were unable to find any changes at the nucleotide level in the promoter regions of *Cyp51A1, Cyp51A2*, and *Cyp51B* of a strain overexpressing these genes, when compared to a wild-type. An overexpressing *Cyp51* phenotype that cannot be linked to promoter changes has previously been reported in azole-resistant isolates of some fungal pathogens including *A. fumigatus* and *Villosiclava virens* (Arendrup et al., 2010; Wang et al., 2015). Future studies of possible *trans*-regulatory changes due to mutations in transcription factors, and analyses of the DNA methylation and histone modification of the promoters may elucidate whether such regulatory elements could be involved in the overexpression of these genes in *Ptt*.

Interestingly, although *Ptt* shares the same host as *P. teres* f. sp. *maculata* (*Ptm*), the other *forma specialis* responsible for SFNB and the predominant form of net blotch disease in Australia (McLean et al., 2010; Murray and Brennan, 2010), azole resistance in this pathogen has not been reported yet. This can probably be explained by the comparatively lower exposure of *Ptm* to DMI fungicides through time. Until comparatively recently, *Ptt* was the predominant net blotch form in Western Australia. *Ptm* was first reported in southern Western Australia in 1995, having previously only been found in the northern wheatbelt. *Ptm* appears to have spread in south-west WA from that time aided by the introduction of stubble retention practices and SFNB sensitive varieties including Franklin (1989), Gairdner (1998), and Baudin (2002) (Paynter et al., 1999; Paynter and Fettell, 2011; Gupta et al., 2012). The prevalence of these varieties in high rainfall areas together with treatment with significant levels of DMI fungicides (Geoff Thomas, personal communication) may underlie the emergence of resistance in *Ptt* first. However, current coexistence of *Ptt* and *Ptm* raises the possibility of fungicide resistance transfer to *Ptm* by rare hybridization events (McLean et al., 2014). A more likely scenario, however, is independent acquisition of resistance by *Ptm* under the same selection pressures. In view of the results presented here, there is an urgent need for adequate anti-resistance management strategies in both types of net blotch disease.

The evolution of new DMI resistant strains in *Ptt* would provide additional challenges to the barley industry. Improved knowledge of fungicide resistance evolution and of the molecular mechanisms by which this occurs will be necessary to implement suitable control strategies that will reduce the likelihood of fungicide resistance outbreaks.

## Author contributions

FL designed the project. WM, RO, WD, and SE contributed to the conception of the work. WM and NB performed the experiments. WM, WD, and PW performed all the bioinformatics analyses. JM and SW performed all the analysis and interpretation of structural modeling data. WM, WD, PW, RO, JM, SW, NB, SE, and FL wrote and edited the manuscript.

### Conflict of interest statement

The authors declare that the research was conducted in the absence of any commercial or financial relationships that could be construed as a potential conflict of interest.
