# Supplementary material for: Demethylase Inhibitor Fungicide Resistance in Pyrenophora teres f. sp. teres Associated with Target Site Modification and Inducible Overexpression of Cyp51
Source: Front Microbiol. 2016 Aug 19;7:1279. doi: 10.3389/fmicb.2016.01279 (PMC4990540; doi:10.3389/fmicb.2016.01279)
Supplement: Supplementary file 1 [file Table1.DOCX]

**Table S1. EC_50_ values of *Pyrenophora teres* f. sp. *teres*** **isolates to boscalid and azoxystrobin**

|  | **EC_50_ (µg mL^-1^)** | |
| --- | --- | --- |
| **Isolate** | **Boscalid** | **Azoxystrobin** |
| **Ko103** | 0.08 (±0.02)^a^ | 0.012 (±0.002) |
| **Ko309** | 0.13 (±0.03) | 0.008 (±0.003) |
| **9179** | 0.09 (±0.02) | 0.011 (±0.002) |
| **9238** | 0.11 (±0.03) | 0.005 (±0.002) |
| **9241** | 0.07 (±0.03) | 0.008 (±0.001) |
| **9264** | 0.06 (±0.01) | 0.011 (±0.004) |
| **10914** | 0.09 (±0.02) | 0.010 (±0.002) |
| **U9** | ND^b^ | 0.007 (±0.001) |
| **Ko310** | 0.11 (±0.02) | 0.015 (±0.005) |
| **Ko603** | 0.13 (±0.02) | 0.004 (±0.002) |
| **9193** | 0.06 (±0.01) | 0.007 (±0.002) |
| **9254** | 0.10 (±0.01) | 0.009 (±0.002) |

EC_50_ values are the mean of at least two independent experiments. ^a^(± Standard error of the mean). ^b^ND=Not determined
