# Supplementary material for: Demethylase Inhibitor Fungicide Resistance in Pyrenophora teres f. sp. teres Associated with Target Site Modification and Inducible Overexpression of Cyp51
Source: Front Microbiol. 2016 Aug 19;7:1279. doi: 10.3389/fmicb.2016.01279 (PMC4990540; doi:10.3389/fmicb.2016.01279)
Supplement: Supplementary file 2 [file DataSheet1.DOCX]

**
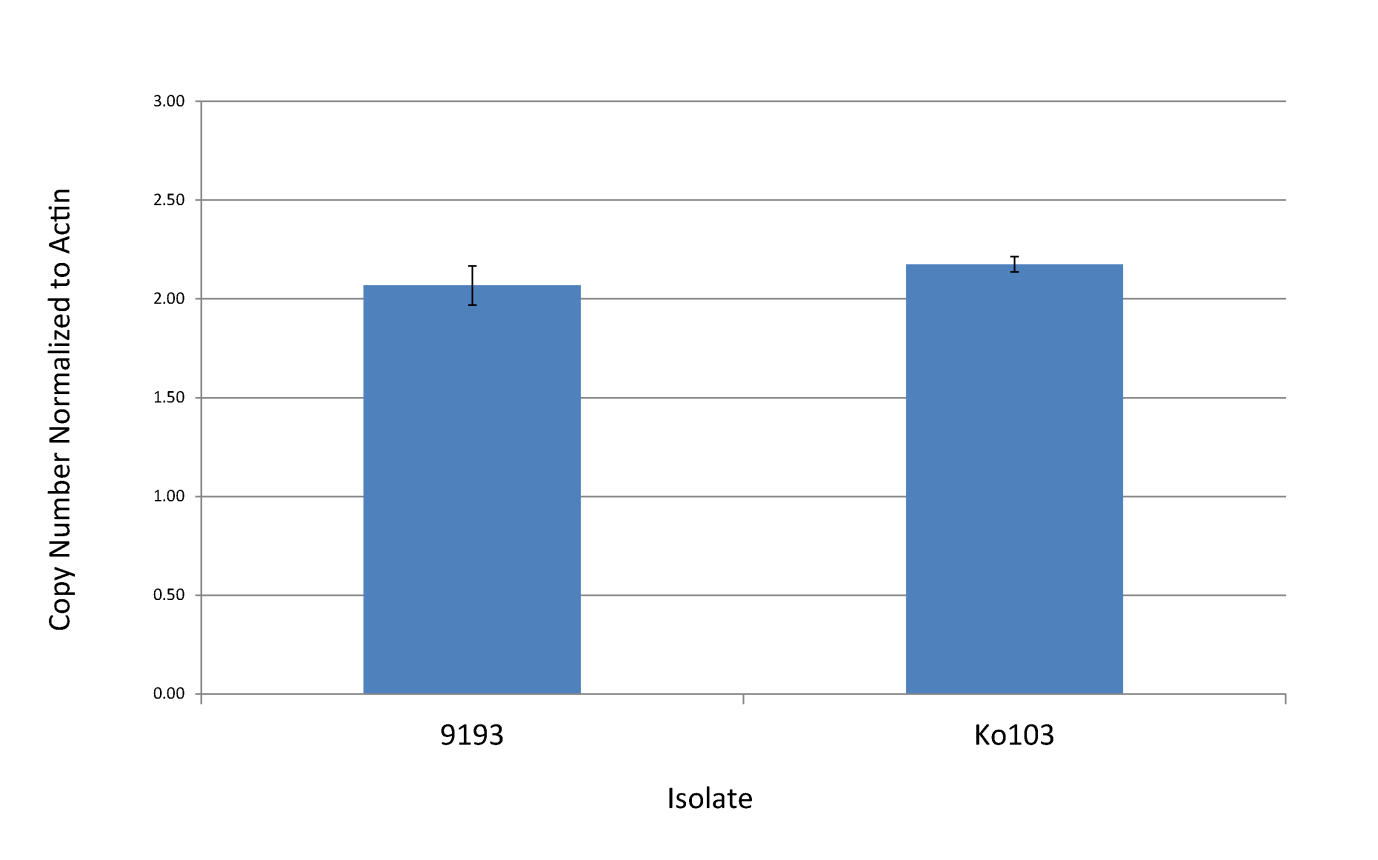
**

**Figure S2. *Cyp51A* copy number in *Pyrenophora teres* f. sp. *teres*.** Histogram shows the number of copies of each gene in the isolates Ko103 and 9193 normalised to that of the single copy gene *Actin* (Genbank accession no XM_003298028). Mean copy number was determined to be 2.17 (SE±0.04) for Ko103 and 2.04 (SE±0.10) for 9193. Standard error bars are shown.
