# Supplementary material for: Demethylase Inhibitor Fungicide Resistance in Pyrenophora teres f. sp. teres Associated with Target Site Modification and Inducible Overexpression of Cyp51
Source: Front Microbiol. 2016 Aug 19;7:1279. doi: 10.3389/fmicb.2016.01279 (PMC4990540; doi:10.3389/fmicb.2016.01279)
Supplement: Supplementary file 3 [file DataSheet2.DOCX]

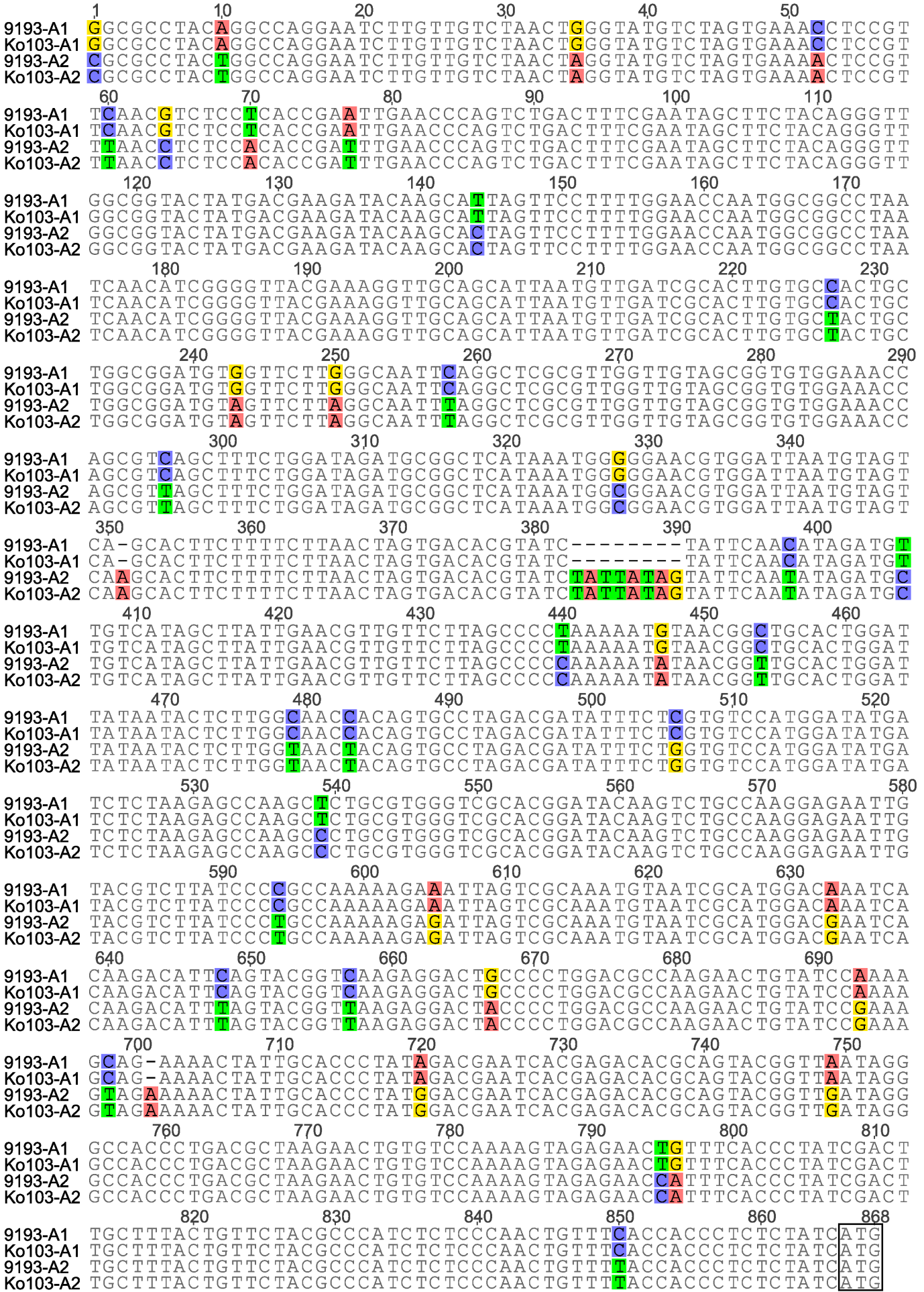


**Figure S3.** Alignment of promoter region of *Cyp51A1* and *Cyp51A2* in isolates 9193 and Ko103. Alignment of a region 864 bp upstream of the start codons of both the *Cyp51A* genes from isolates 9193 and Ko103. Polymorphisms in the promoter region are highlighted, start codons are boxed. Alignment generated in Geneious version 6.1 software (Biomatters) using ClustalW algorithm with IUB scoring matrix, gap opening penalty 15, gap extension penalty 6.66.
